# Dengue emergency in the Americas: time for a new continental eradication plan

**DOI:** 10.1016/j.lana.2023.100539

**Published:** 2023-06-15

Incidence of dengue has grown dramatically in the past 20 decades, with global cases jumping from 500,000 in 2000 to 5.2 million in 2019, the largest number of dengue cases ever reported. Of these, 3.1 million were recorded in the region of the Americas, spreading from south USA to northern Argentina. In the first 5 months of 2023, the region has already reported 1.9 million cases, more than in the whole of 2021 and projected to surpass the 2.8 million registered in 2022. Besides the increased health burden and deaths, what is concerning about the current epidemic is the geographical expansion of cases beyond the historically endemic areas, affecting unprepared regions without vector control programmes in place and with health systems not trained to diagnose and manage the disease. Hitting the region just as it starts to recover from the COVID-19 pandemic, the dengue epidemic should not be overlooked.

Argentina is going through the deadliest dengue outbreak in its history, with over 60 deaths and more than 100,000 cases. Bolivia and Peru, although considered endemic, are also facing record-breaking case numbers. According to the Pan American Health Organization, both countries have passed 110 ,000 cases reported. With an overwhelmed health system, Bolivia reported a high number of deaths among children, in what is now the worst dengue epidemic in the past 10 years. In Peru, not only are the numbers high, but also the epidemiology of the disease has changed. Historically, case numbers in the country are driven by the Amazonian regions, but the 2023 outbreak was heavily felt in Lima. The county's capital registered a >500% increase in cases compared with the same epidemiological weeks of 2021, and a 14-times increase compared with 2017, the year Peru experienced its worst epidemic. With a National Health Emergency in place since February, the number of cases in the first 4.5 months of 2023 had already surpassed the 2017 total. One possible compounding factor for this is the highly inefficient water system in Peru. Limited flowing water in peri-urban areas, longer hot-seasons, and heavy rains from El Niño likely contributed to more standing water, an increased mosquito population, and the current outbreak.

Despite being a threat to more than 100 countries, dengue is restricted to areas where its vector, the *Aedes* mosquito, can adapt and thrive. *Aedes* is a highly adaptable species and the world has seen a rapid expansion of dengue cases, driven by the migration and adaptation of the vector and strongly related to the warming of previously temperate climates such as southern Europe and the USA and South America's southern cone. In a comment published in August 2022, Codeco and colleagues called attention to this issue, “The expansion [of dengue] in the Southern frontier [of Brazil] suggests increased climate suitability for *Aedes* mosquitoes and risk of vector-borne diseases co-circulating between Argentina–Paraguay–Brazil”.

Although the mosquito is essential for transmission, the establishment of the virus within the population should also be factored in. One study that screened mosquitoes after the 2022 outbreak in Miami-Dade, FL, USA, suggested silent circulation of the virus in the state—contrasting with the historical assumption of imported sources in previous outbreaks. In the accompanying comment, authors highlight the compounding threat of mosquito adaptation and subclinical cases in the geographical expansion of dengue, allowing for the establishment of permanent hotspots and new endemic areas.

In the 1940s and 50s, a yellow fever focused programme successfully eradicated *Aedes aegypti* in Brazil using a DDT-based strategy but relaxation of the strategies allowed for the reintroduction of the mosquito soon after. Luckily, several emerging strategies can help in this fight. Several techniques to create and release sterile mosquitos to control the vector population have been tested in Brazil using *Wolbachia*-infected and genetically-modified *Aedes*. A large, ongoing field experiment in Medellin, Colombia, has released 40 million *Wolbachia*-infected *Aedes* mosquitos every week since 2015 and, according to Professor Iván Darío Vélez (manager of the Colombian branch of the World Mosquito Program [WMP]), an 89% reduction in infections was observed since the project started. To counteract the current outbreak, thousands of DNA-damaged male mosquitos were released in the most-affected districts of Argentina. Vector-modifying strategies control breeding of mosquitos without eliminating the population, thus maintaining an environmental balance and being preferred over mass-insecticide approaches. Despite the logistical limitations of production, storage, and deployment of millions of mosquitos, the cost-effectiveness of such strategies has been demonstrated in other countries. In March, the Brazilian Government announced a partnership with the WMP to build a mosquito biofabric with the capacity to breed up to 100 million mosquitoes per week and the potential to dramatically expand the use of modified mosquitos across Brazil. As for virus control, in Argentina, the health regulatory agency authorised the use of Takeda's vaccine, TAK-003 (Qdenga), for adults and children older than 4 years. The vaccine, which was also approved in Brazil, could be an important step for the long-term elimination of dengue in the region; however, there is no local production and deployment is dependent on importation times and prices. As we've seen with COVID-19, reliance on outsourced vaccine production can be disastrous for a health emergency, and locally or regionally produced vaccines free of intellectual property barriers will be key to the success of this strategy in the region.

The current dengue emergency might be the result of a perfect storm, with favourable climate conditions resulting from global warming, reduced surveillance in endemic areas that redirected preventive programmes to COVID-19, a possible increase in testing and diagnosis due to similar symptoms as COVID-19, and the overlapping of a new epidemic with a tired, dismantled health-care system after COVID-19. However, it is unlikely that the 2023 epidemic is an outlier. Dengue control and elimination will require a multisectoral strategy with government-led programmes to target vector control and virus circulation and community-focused actions including provision of piped water, educational programmes to reduce available breeding sources, and training of health-care workers to better diagnose and manage cases.

Dengue and other vector-borne diseases will continue to expand throughout the region (and the globe) as climate change progresses, and it is imperative for countries to prepare for the evolving situation and develop national and regional strategies to contain the advance of the disease. In 1947, PAHO accepted a joint proposition from Argentina, Bolivia, Brazil, and Paraguay for a *Continental Aedes Aegypti* eradication plan, acknowledging that the mosquito sees no borders and a unified strategy was required. Now it is time to renew those vows.

